# Advancements in Radiogenomics for Clear Cell Renal Cell Carcinoma: Understanding the Impact of BAP1 Mutation

**DOI:** 10.3390/jcm13133960

**Published:** 2024-07-06

**Authors:** Federico Greco, Valerio D’Andrea, Andrea Buoso, Laura Cea, Caterina Bernetti, Bruno Beomonte Zobel, Carlo Augusto Mallio

**Affiliations:** 1 Department of Radiology, Cittadella della Salute, Azienda Sanitaria Locale di Lecce, Piazza Filippo Bottazzi, 2, 73100 Lecce, Italy; 2 Research Unit of Radiology, Department of Medicine and Surgery, Università Campus Bio-Medico di Roma, Via Alvaro del Portillo, 21, 00128 Roma, Italy; valerio.dandrea@unicampus.it (V.D.); andrea.buoso@unicampus.it (A.B.); laura.cea@unicampus.it (L.C.); c.bernetti@policlinicocampus.it (C.B.); b.zobel@policlinicocampus.it (B.B.Z.); c.mallio@policlinicocampus.it (C.A.M.); 3 Fondazione Policlinico Universitario Campus Bio-Medico, Via Alvaro del Portillo, 200, 00128 Roma, Italy

**Keywords:** radiogenomics, radiomics, texture analysis, renal cancer, clear cell renal cell carcinoma, BAP1

## Abstract

Recent advancements in understanding clear cell renal cell carcinoma (ccRCC) have underscored the critical role of the BAP1 gene in its pathogenesis and prognosis. While the von Hippel–Lindau (VHL) mutation has been extensively studied, emerging evidence suggests that mutations in BAP1 and other genes significantly impact patient outcomes. Radiogenomics with and without texture analysis based on CT imaging holds promise in predicting BAP1 mutation status and overall survival outcomes. However, prospective studies with larger cohorts and standardized imaging protocols are needed to validate these findings and translate them into clinical practice effectively, paving the way for personalized treatment strategies in ccRCC. This review aims to summarize the current knowledge on the role of BAP1 mutation in ccRCC pathogenesis and prognosis, as well as the potential of radiogenomics in predicting mutation status and clinical outcomes.

## 1. Introduction

In recent years, significant research efforts have been directed towards identifying clinical and pathological characteristics linked to the prognosis of patients with clear cell renal cell carcinoma (ccRCC), particularly within the framework of emerging targeted therapies [1].

The majority of genomic information from the Cancer Genome Atlas Program is ccRCC, which stands as the most widespread subtype of renal cell carcinoma (RCC) [2].

The most prevalent mutation in ccRCC is the von Hippel–Lindau (VHL) mutation [3]. However, VHL mutation does not hold prognostic value [4]. A recent comprehensive characterization of ccRCCs has identified other genomic alterations, such as polybromo-1 (PBRM1), BRCA1-associated protein 1 (BAP1), SET domain containing 2 (SETD2), and lysine-specific demethylase 5C (KDM5C), and unlike VHL, these genetic alterations have been linked to patient prognosis or survival, leading to increased interest in radiogenomics in ccRCCs [5]. BAP1, a nuclear deubiquitinase, is inactive in nearly 15% of ccRCCs [6]. Studies have indicated that BAP1 mutation serves as an unfavorable predictive factor for overall survival in patients with ccRCC [5].

Radiogenomics, a branch of radiology that explores the correlation between the imaging characteristics of a disease and its gene expressions and mutations, has experienced significant growth in recent years, prompting researchers to identify quantitative and qualitative imaging markers that correlate with genetic and histological characteristics, enhancing pathological analysis [2,7,8,9].

On the other hand, texture analysis represents a quantitative image processing technique used to detect repetitive patterns that may not be discernible to the human eye [10].

Texture analysis, radiogenomics, and advancements specific to imaging modalities have produced a range of biomarkers for RCC in research. While numerous methods show promise, it is crucial to standardize and validate these procedures before integrating them into clinical practice [11].

The purpose of this review is to present the current knowledge of radiogenomics of ccRCC, underlying the phenotypic expression of BAP1 mutation, assessed via retrospective analyses.

## 2. Role of BAP1 in Clear Cell Renal Cell Carcinoma

The BAP1 gene plays a crucial role in the pathogenesis of ccRCC, a subtype of kidney cancer characterized by distinct molecular alterations.

BRCA1-related protein 1—BAP1 gene is a cancer suppressor found on human chromosome 3p21.3 and codes for ubiquitin carboxy-terminal hydrolase [12].

BAP1 functions as a vital tumor suppressor gene in various cancers, including melanoma, mesothelioma, and RCC, being mutated both somatically and in the germline [13]. It comprises 17 exons, and encodes a 729 amino acid of 90 kDa protein. This protein, operating as a deubiquitinating enzyme, features an N-terminal ubiquitin carboxy-terminal hydrolase (UCH) domain, a host cell factor 1 binding motif (HBM), and a C-terminal domain interacting with ASXL1/2, localizing in the nucleus via a nuclear localization signal (NLS) in the C-terminus [13,14,15].

The protein is a deubiquitinating enzyme that interacts with breast cancer type 1 susceptibility protein (BRCA1) and BRCA1-associated RING domain protein 1 (BARD1) proteins, inhibiting their ubiquitination capacity and acting as a tumor suppressor [16]. The region of BAP1 that binds to BRCA1 spans amino acids 596–721, while the region that interacts with BARD1 is from 182 to 365 [1].

Moreover, BAP1 modulates histone deubiquitination, influencing transcription, cell cycle progression, and DNA damage response, is predominantly localized in the nucleus, and plays a pivotal role in regulating the G1/S phase transition [17].

Particularly in ccRCC, BAP1 acts as a two-hit tumor suppressor, and the loss of both alleles of BAP1, typically through mutations, contributes to tumorigenesis [6]. BAP1 mutations can affect its enzymatic activity and protein interactions, thereby shaping its tumor-related functions, including those in RCC [6].

Understanding the intricate molecular mechanisms underlying BAP1’s activity sheds light on its significance in both normal cellular processes and cancer development, particularly in RCC [1].

A recent comprehensive review [12] highlights the consistent correlation between BAP1 changes and unfavorable outcomes in RCC patients, underscoring the need for prospective studies to assess their predictive relevance, particularly in immunotherapy response [12]. This necessitates a multidimensional approach integrating various biomarkers to enhance predictive and prognostic accuracy in RCC management.

A clinicopathologic analysis [18] of 14 molecularly confirmed tumors revealed that BAP1-mutated ccRCC commonly displays typical histological features, such as papillary structure, eosinophilic cytoplasm, and cytoplasmic granules. Moreover, BAP1-mutated ccRCCs typically exhibit expression of p504S and CK7, unlike the majority of ccRCCs, accompanied by almost universal loss of nuclear BAP1 expression-detected immunohistochemically, and their study group exhibited a notable incidence of renal vein involvement, stage pT3 disease, and metastasis [18].

## 3. Material and Methods

### 3.1. Search Strategy

A narrative literature research was conducted in March 2024 utilizing electronic databases: PubMed, Cochrane Library, and Scopus. The search terms employed included: (radiogenomics OR radiomics OR texture analysis) AND (clear cell renal cell carcinoma OR renal cancer) AND BAP1. Additionally, a manual search was performed using Google Scholar, and the reference lists of all eligible articles identified through the database search were screened. No limitations were imposed on the timeframe for the literature search.

After removing duplicates, the titles and abstracts of the remaining articles underwent screening to assess their alignment with the selection criteria. To further validate eligibility, the full texts of the remaining articles were scrutinized using inclusive standards.

The inclusion criteria consisted of articles published in the English language, without limitations on the timeframe, and addressing the topic of radiogenomics of ccRCC. Exclusion criteria included publications not available in English, studies not related to ccRCC or radiogenomics, duplicate articles, and non-primary literature such as abstracts and letters to the editor. Two independent authors (VD and FG) initially screened abstracts and then assessed full-text articles.

### 3.2. Data Extraction

The following information was systematically extracted: author and year of publication, study design, number of study participants, gender and age of participants, imaging technique utilized, genes studied, study results, and with/without texture analysis.

All the eight studies included in our literature review are summarized in Table 1.

## 4. Discussion

As new technologies like artificial intelligence and genome sequencing advance rapidly, radiogenomics has emerged as a cutting-edge field in personalized medicine [26]. Furthermore, the integration of biomedical research with genomics has been facilitated by the availability of open-source data from the Human Genome Project. Several investigations cited in this review relied on TCGA, a comprehensive repository documenting genetic alterations across various cancer types, including RCC. All data, spanning from medical images to tissue samples from TCGA, are linked to a unified identifier and are freely accessible for download [3,9,27].

Regarding BAP1 mutation, three radiogenomics studies have been selected with different outcomes and statistically significant results [19,20,21].

Karlo et al. [19] explored the relationship between imaging characteristics and gene mutations. Mutations in KDM5C and BAP1 genes showed significant associations with renal vein invasion evident on contrast-enhanced CT scans of ccRCC (*p* = 0.022 and 0.046, respectively). The genetic profile of solid ccRCC differed markedly from multicystic ccRCC, with VHL and PBRM1 mutations being more prevalent in solid ccRCC, while SETD2, KDM5C, and BAP1 mutations were entirely absent in multicystic ccRCC. Notably, BAP1 and KDM5C mutations were specifically linked to heightened evidence of renal vein invasion, aligning with previous findings indicating their association with advanced tumor stage, grade, and invasiveness [28,29]. These results underscore the potential genetic basis for phenotypic variations in ccRCC and highlight the importance of further investigation to validate these associations in larger cohorts.

**Figure 1:**
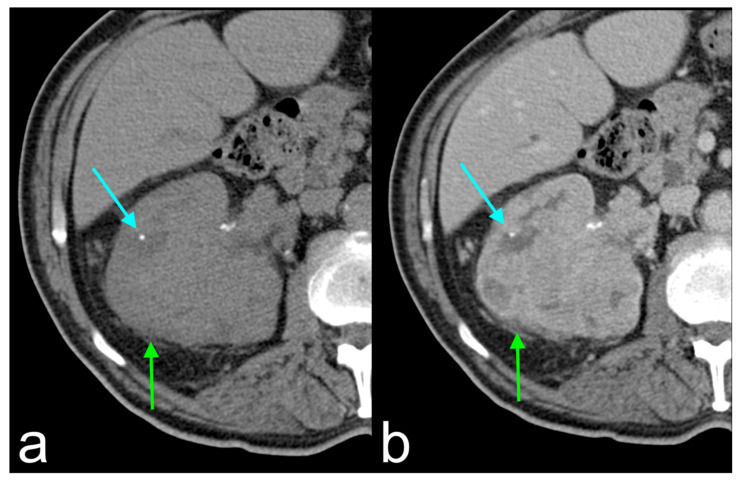
Unenhanced axial CT image (**a**) and axial CT image during venous phase (**b**) showing ccRCC with BAP1 gene mutation with ill-defined margins (green arrows) and tumor calcification (light blue arrows). Images from The Cancer Genome Atlas Kidney Renal Clear Cell Carcinoma Collection (TCGA-KIRC); https://www.cancerimagingarchive.net/collection/tcga-kirc/ (accessed on 1 May 2024).

Shinagare et al. [20] examined similar imaging features as earlier investigations, concentrating on the most common mutations. The study revealed significant associations between certain imaging features and mutations, particularly related to BAP1 and MUC4. An ill-defined margin (*p* = 0.001) and tumor calcification (*p* < 0.001) were linked to the BAP1 mutation, aligning with previous findings correlating such features with aggressive ccRCC (Figure 1). Ill-defined margins often signal locally invasive and aggressive ccRCC, traits commonly seen in high-grade tumors linked to the BAP1 mutation. However, not all poorly demarcated tumors harbored the BAP1 mutation, indicating the need for further investigation into the relationship between imaging features and mutational status. Additionally, while tumor calcifications have not typically been linked to adverse outcomes, their association with the BAP1 mutation warrants deeper exploration. Furthermore, the study hinted at therapeutic implications, suggesting that BAP1 mutation might influence treatment response, potentially guiding treatment decisions [20]. These results underscore the potential of radiogenomics in predicting genomic profiles and clinical outcomes in ccRCC, yet highlight the complexities and challenges in achieving reliable interobserver agreement and integrating imaging as a non-invasive biomarker for mutational status prediction.

Wu et al.’s study [21] partially aligns with these findings, examining the semantic CT features linked to BAP1 and/or TP53 gene mutations. Kaplan–Meier analysis demonstrated a significant association between BAP1 and/or TP53 mutation and poorer survival outcomes. Multivariate logistic regression revealed that certain CT features such as ill-defined margin (*p* = 0.001), spiculated margin (*p* = 0.018), renal vein invasion (*p* = 0.002), and renal pelvis invasion (*p* = 0.001) were independent predictors of BAP1 and/or TP53 mutation. A nomogram was created incorporating these features, achieving a high area under the receiver operating characteristic curve of 0.872. Additionally, a nomogram combining these four CT features was developed for predicting BAP1 and/or TP53 mutation status, suggesting its potential as a reliable method for predicting mutation status in ccRCC [21].

In the realm of radiogenomics, when encountering ccRCC exhibiting ill-defined margins on imaging, a key consideration is the differential diagnosis between BAP1 mutation, P4HA3 expression, ADAM12 expression, and high methylation levels of tumor suppressor RUNX3. Ill-defined margins emerge as a common characteristic among these entities, suggesting a potential overlap in their radiological presentation. However, nuances in imaging features can help differentiate between them. Specifically, while BAP1 mutation-associated ccRCC often presents with tumor calcification and renal vein invasion, P4HA3 expression is more commonly associated with tumor infiltration and advanced tumor stage, as indicated by the American Joint Committee of Cancer (AJCC) staging. On the other hand, ADAM12 expression correlates with larger primary tumor size and tumor necrosis. Moreover, predictive radiogenomic analysis of high methylation levels of tumor suppressor RUNX3 reveals additional considerations, such left-sided tumors, and the presence of intratumoral vascularity. This distinctive array of imaging characteristics offers a nuanced approach to the radiogenomic differential diagnosis of ccRCC, aiding in the precise identification of underlying genetic alterations and epigenetic modifications [7,8,19,20,21,30].

In ccRCC, tumor calcification is often observed as a characteristic feature in cases with BAP1 mutations [19]. Tumor calcification refers to the presence of calcium deposits within the tumor tissue, which can be visualized through radiological imaging techniques such as CT scans. However, despite the association between tumor calcification and BAP1 mutations, there are currently no specific mutations or gene expressions identified that directly lead to the phenotypical expression of this radiological feature in ccRCC.

Texture analysis was investigated in five studies [5,22,23,24,25], all based on the use of CT.

Kocak et al. [5] intended to assess the possible utility of machine learning-based unenhanced CT texture analysis in predicting the BAP1 mutation status of ccRCCs, relying solely on highly dependable texture characteristics. Their study involved 65 ccRCC cases, 13 with and 52 without BAP1 mutation, analyzing texture features extracted from unenhanced CT images through manual segmentation and PyRadiomics software. They employed a Random Forest classifier, achieving an 84.6% accuracy in classifying ccRCCs based on BAP1 mutation status, with an area under curve (AUC) of 0.897 and respectable sensitivity, specificity, and precision values. Specifically, for predicting ccRCCs with BAP1 mutation, their model showed a sensitivity of 90.4% and specificity of 78.8%, with precision of 81% [5]. Their findings suggest a promising predictive capability of the method, with over 80% of ccRCCs successfully classified by BAP1 mutation status. The study’s implications extend to practical and clinical realms, as understanding the molecular genetics of ccRCCs can enhance patient classification, prognosis prediction, and personalized treatment strategies. It is considered plausible that the BAP1 mutation could serve as a notable prognostic indicator (indicating worse prognosis and lower survival rates), potentially assisting in distinguishing between ccRCCs with or without this mutation using texture features derived from unenhanced CT analysis.

In a separate investigation, Chen et al. [22] examined a group of 57 ccRCC patients to predict the status of VHL, PBRM1, and BAP1 mutations, employing contrast-enhanced CT scans. Instead of employing a singular classifier, the authors introduced a novel multi-classifier multi-objective (MCMO) radiogenomics predictive model, which attained AUC values > 0.85 for predicting each mutation. Three specific characteristics (Variance, Sum average, and Sum variance) related to the BAP1 gene displayed *p*-values above 0.05. The multi-classifier multi-objective (MCMO) approach outperformed individual classifiers, exhibiting superior AUC, accuracy, sensitivity, and specificity. Notably, MCMO achieved a prediction accuracy of 0.81, 0.78, and 0.90 for VHL, PBRM1, and BAP1 genes, respectively, with AUC values of at least 0.86 and sensitivity and specificity exceeding 0.75 and 0.80, respectively [22].

The predominant features of BAP1 included one geometric characteristic, one intensity feature, and four texture features [22]. Texture attributes notably influenced the predictive model for BAP1. Differently from Shinagare et al. [20], tumor margins were not examined due to their qualitative nature. However, calcification presence might correlate with specific features like Homogeneity and Variance [22].

In accordance with the precedent models, Feng et al. [23] state that CT radiomics represents a promising and viable approach for predicting BAP1 mutation status in ccRCC patients with outstanding performance in the TCGA dataset. The texture features of tumor images were extracted using the Matlab-based IBEX package. A model for predicting BAP1 mutation status was built using Random Forest Classification algorithms and assessed using leave-one-out cross-validation. The Random Forest model for predicting BAP1 mutation status achieved an accuracy of 0.83, sensitivity of 0.72, specificity of 0.87, precision of 0.65, AUC of 0.77, and an F-score of 0.68 [23]. Their exploration could carry more tangible and clinical importance when compared to earlier studies. Among the top four frequently altered genes, BAP1 stands out as particularly crucial for personalized target therapies.

In relation to prior studies on imaging research of BAP1 mutation using TCGA and TCIA data, Ghosh et al. [24] discovered that the predictive model relying on nephrographic phase images exhibited the highest performance, achieving an AUC of 0.71. However, they neglected to implement appropriate modifications when the count of BAP1 mutations was insufficient. Their investigation highlights that CT texture analysis and summary measures on pre-therapy baseline images are predictive of BAP1 mutation status in patients with ccRCC. These measures were derived from standard diagnostic CT scans, suggesting that such image-derived techniques, as described, can be easily replicated with minimal additional cost and have potential applicability for widespread use as non-invasive biomarkers for other diseases [24].

Finally, consistent with the previous studies, Zeng et al. [25] showed that the BAP1 mutation was effectively identified using radiomics features in patients with ccRCC. Using CT image analysis, the random forest algorithm demonstrated good capacity to identify the BAP1 mutation, with an AUC of 0.955. Moreover, radiomics models showed promising prognostic value for overall survival (OS) in validation sets, with a 5-year AUC of 0.775. Integrating radiomic data with omics further improved predictive accuracy, with a 0.846 AUC for the multi-omics model. Presence of the BAP1 mutation correlated with poorer prognosis, underscoring the importance of radiomic features in risk assessment and personalized treatment of ccRCC patients [25].

Our literature review has some limitations. First and foremost, all studies selected in our literature review are retrospective studies; therefore, further prospective studies are desirable in the future. Secondly, the size of our patient cohort was relatively small and unbalanced because of the rarity of ccRCCs with BAP1 mutation; thus, multicenter studies and large-scale cohorts are needed. Thirdly, the imaging data from TCGA-KIRC in TCIA encompasses patients from diverse centers/sources, which could be viewed as a significant limitation due to variations in acquisition protocols. Last but not least, the emphasis of previous studies was mainly on CT imaging features, with Shinagare et al. [20] delving into magnetic resonance imaging (MRI) features to a limited degree, analyzing data from 22 out of 103 patients in their study. MRI is attracting interest due to its radiation-free nature and its capability to extract supplementary information from diverse sequences such as T2-weighted and diffusion-weighted imaging. These attributes could enrich the development of image prediction models by integrating radiogenomic features. Further investigation into MRI features in upcoming studies shows potential for deepening our comprehension of radiogenomic associations.

## 5. Advantages of Radiogenomics

Combining imaging phenotypes with molecular processes is crucial for understanding tumor biology and analyzing intrinsic tumor heterogeneity and characteristics, which have significant implications for patient care, improving diagnostic precision. A key advantage of radiogenomics is its ability to provide data that are not always accessible through genomic tests on biopsy specimens. This is because gene expression and mutations are analyzed on small samples, rather than the entire tumor lesion, potentially missing the overall heterogeneity of the disease, which is common in ccRCC [31]. Radiogenomics is of fundamental importance to prepare a targeted therapeutic plan that can attack the tumor with specific mutation and/or gene expression and to avoid certain non-invasive therapies’ responsiveness. Specifically, a study has suggested that BAP1 mutations may be linked to markers indicating responsiveness to immune checkpoint inhibitors, since BAP1 mutations are more frequently observed in sarcomatoid and rhabdoid tumors, which are known to respond better to immunotherapy drugs [32]. Radiogenomics has broad applications in oncological pathology across various types of cancer. It allows for the detection of gene expression or mutations through a non-invasive approach, enabling the application of personalized therapies [26]. For example, identifying the Kirsten rat sarcoma virus (KRAS) mutation in colorectal cancer serves as a downstream marker of tumor resistance to anti-epidermal growth factor receptor (EGFR)-targeted therapy. Additionally, non-small cell lung cancer (NSCLC) with anaplastic lymphoma kinase (ALK) rearrangement is more responsive to ATP-competitive ALK inhibitors such as crizotinib, ceritinib, and alectinib [26].

Radiogenomics facilitates the tracking of tumor evolution and treatment response over time through repeated imaging. Furthermore, the acquisition of genomic data using a non-invasive approach does not expose the patient to possible complications related to the biopsy procedure. Lastly, it can be more cost-effective than extensive genomic testing, especially when repeated evaluations are necessary.

Overall, radiogenomics integrates imaging and genomic data to offer a more holistic and detailed understanding of tumor biology, which can lead to better patient outcomes through tailored diagnostic, prognostic, and therapeutic approaches.

## 6. Conclusions

Our review underscores the critical role of the BAP1 gene in ccRCC pathogenesis, highlighting its importance as a prognostic factor. Numerous studies have established the correlation between BAP1 mutations and unfavorable prognoses in ccRCC patients, suggesting the potential of BAP1 mutation status in guiding treatment strategies and prognostic assessments. Radiogenomic approaches, including both textured and textureless analyses, offer promising avenues for predicting BAP1 mutation status and overall survival outcomes in ccRCC patients. Leveraging radiogenomics provides an advantageous tool in identifying BAP1 mutations, facilitating more personalized and informed clinical decisions. Nevertheless, further prospective studies involving larger cohorts and standardized imaging protocols are imperative for validating these findings and effectively integrating them into clinical practice.

## Figures and Tables

**Table 1:** Summary of radiogenomics and texture analysis studies.

Author/Year	Type of Study: Patient (n)	Imaging Technique	Genes	Outcomes Related with Bap Mutation	With/without Texture Analysis
**Karlo et al., 2014 [19]**	Retrospective: 233	CT	VHL, **BAP1**, KD5MC	Mutations of KDM5C and **BAP1** were significantly associated with evidence of renal vein invasion (*p* = 0.022 and 0.046, respectively). The genotype of solid ccRCC differed significantly from the one of multicystic ccRCC. While mutations of SETD2, KDM5C, and **BAP1** were absent in multicystic ccRCC, mutations of VHL (*p* = 0.016) and PBRM1 (*p* = 0.017) were significantly more common among solid ccRCC.	**Without**
**Shinagare et al., 2015 [20]**	Retrospective: 103	CT/MR	VHL, **BAP1**, PBRM1, SETD2, KDM5C, and MUC4	**BAP1** mutation was associated with ill-defined tumor margins and presence of calcification (*p* = 0.02 and 0.002, respectively, Pearson’s χ2 test); MUC4 mutation was associated with exophytic growth (*p* = 0.002, Mann–Whitney *U* test).	**Without**
**Wu et al., 2022 [21]**	Retrospective: 156 patients	CT	**BAP1**, TP53	Kaplan–Meier analysis revealed a significant correlation between **BAP1** and/or TP53 mutation and poorer survival outcomes. Multivariate binary logistic regression analysis identified ill-defined margin (*p* = 0.001), spiculated margin (*p* = 0.018), renal vein invasion (*p* = 0.002), and renal pelvis invasion (*p* = 0.001) as independent predictors of **BAP1** and/or TP53 mutation. A nomogram containing these 4 semantic CT features was constructed; the area under the receiver operating characteristic curves was 0.872 (95% CI, 0.809–0.920). **BAP1** and/or TP53 mutation were significantly associated with advanced American Joint Committee on cancer stage (stage III–IV, *p* = 0.002), a higher (WHO/ISUP) grade (G3-4, *p* = 0.032), and higher T stage (T3-4, *p* = 0.03).	**Without**
**Kocak et al. [5]**	Retrospective: 65 patients (13 with and 52 without **BAP1** mutation).	Unenhanced CT	**BAP1**	No statistically difference for tumor size *p* = 0.517 and CT attuenation *p* = 0.838 between ccRCCs with and without **BAP1** mutation. The RF classifier accurately categorized 84.6% of the ccRCCs based on **BAP1** mutation status, achieving an AUC value of 0.897. The weighted average sensitivity, specificity, and precision stood at 84.6%, 84.6%, and 85.1%, respectively. In predicting ccRCCs with **BAP1** mutation, the sensitivity, specificity, and precision were 90.4%, 78.8%, and 81%, respectively.	**With:** CT acqusition parameters: slice thickness, kV, mAs, pixel size. Texture feature extracted by PyRadiomics software (Python 2.7.13; PyRadiomics 2.0.1; Numpy 1.13.1; SimpleITK 1.1.0; PyWavelet 0.5.2). Mann–Whitney U test was used for comparison of tumor size and CT attenuation. Feature selection was performed using WEKA toolkit version 3.8.2. RF was used for model development.
**Chen et al., 2018 [22]**	Retrospective: 57	CT	VHL, PBRM1, **BAP1**	Using the proposed MCMO model, they achieved a predictive area under the receiver operating characteristic curve (AUC) over 0.85 for VHL, PBRM1, and **BAP1** (AUC = 0.955) genes with balanced sensitivity and specificity.	**With:** They proposed a MCMO radiogenomics predictive model.
**Feng et al., 2020 [23]**	Retrospective: 54 patients	CT	**BAP1**	The results indicate that the RF-based predictive model demonstrated an accuracy of 0.83 [95% CI: 0.76–0.88], a sensitivity of 0.72 (95% CI: 0.65–0.79), a specificity of 0.87 (95% CI: 0.82–0.93), a precision of 0.65 (95% CI: 0.58–0.74), an AUC of 0.77 (95% CI: 0.70–0.83), an F-score of 0.68 (95% CI: 0.61–0.76), and an MCC of 0.58 (95% CI: 0.50–0.66).	**With:** texture features extracted with Matlab-based IBEX package. They used SMOTE to analyze and stimulate data and LOCCV for cross validation.
**Ghosh et al., 2015 [24]**	Retrospective: 78 patients	CT	**BAP1**	The subset of 4037 nephrographic features yielded the most favorable adjusted *p* values, indicating their significant predictive ability for **BAP1** mutation status. No significant features with adjusted *p*-values ≤ 0.1 were identified from other phases of renal CT scans. The RF classifier, trained to forecast gene mutation status from 3-D texture features, achieved AUC values of 0.66 (nc), 0.62 (cm), 0.71 (neph), and 0.52 (ex), respectively, for **BAP1** mutation.	**With:** molecular data regarding gene mutation status were sourced from the cBioPortal. The correlation between image features and gene mutation status was evaluated utilizing the Mann–Whitney–Wilcoxon rank-sum test. Additionally, the random forests classifier in the Waikato Environment for Knowledge Analysis (WEKA) software was employed to evaluate the predictive capability of computationally derived image features in distinguishing cases with **BAP1** mutations for ccRCC.
**Zeng et al., 2021 [25]**	Retrospective: 207	CT	VHL, **BAP1**, PBRM1, SETD2, molecular subtypes (m1–m4)	They evaluated the potential value of CT radiomics features in classifying mutations and molecular subtypes of ccRCC, with the use of multiple machine learning algorithms. Leveraging radiomics features, the random forest algorithm exhibited strong capability in detecting mutations in VHL (AUC = 0.971), **BAP1** (AUC = 0.955), PBRM1 (AUC = 0.972), and SETD2 (AUC = 0.949), as well as molecular subtypes m1 (AUC = 0.973), m2 (AUC = 0.968), m3 (AUC = 0.961), and m4 (AUC = 0.953).	**With:** they employed 4 algorithms (GBDT, LASSO, RF, XGBoost) for feature selection and 8 algorithms (RF, GBDT, AdaBoost, LR, DT, SVM, NB, KNN) as classifiers.

Abbreviations: AdaBoost, adaptive boosting; AUC, area under curve; BAP1, BRCA-1 Associated Protein-1; ccRCC, clear cell renal cell carcinoma; CU, confidence intervals; CT, computed tomography; DT, decision tree; GBDT, gradient boosting decision tree; ISUP, International Society of Urologic Pathology; KDM5C, lysine-specific demethylase 5C; KNN, K-nearest neighbor; LASSO, least absolute shrinkage and selection operator; LOCCV, leave-one-out-cross-validation; MCC, Matthews correlation coefficient; LR, logistic regression; MCMO, multi-classifier multi-objective; MR, magnetic resonance; MUC4, mucin-4; NB, naive Bayesian; PBRM1, protein polybromo-1; RF, random forest; SETD2, SET domain containing 2; SMOTE, synthetic minority over-sampling technique; SVM, support vector machine; TP53, tumor protein P53; VHL, von Hippel–Lindau; WEKA, Waikato Environment for Knowledge Analysis; WHO, World Health Organization; XGBoost, extreme gradient boosting.
